# Pharmacokinetic Aspects of Nanoparticle-in-Matrix Drug Delivery Systems for Oral/Buccal Delivery

**DOI:** 10.3389/fphar.2019.01057

**Published:** 2019-09-24

**Authors:** Renata Carvalho Feitosa, Danilo Costa Geraldes, Viviane Lucia Beraldo-de-Araújo, Juliana Souza Ribeiro Costa, Laura Oliveira-Nascimento

**Affiliations:** ^1^Laboratory of Pharmaceutical Technology (Latef), Faculty of Pharmaceutical Sciences, State University of Campinas, Campinas, Brazil; ^2^Institute of Biology, State University of Campinas, Campinas, Brazil

**Keywords:** nanoparticle, oral delivery, buccal delivery, matrix delivery, drug absorption

## Abstract

Oral route maintains its predominance among the ones used for drug delivery, especially when medicines are self-administered. If the dosage form is solid, therapy gains in dose precision and drug stability. Yet, some active pharmaceutical substances do not present the required solubility, permeability, or release profile for incorporation into traditional matrices. The combination of nanostructured drugs (nanoparticle [NP]) with these matrices is a new and little-explored alternative, which could bring several benefits. Therefore, this review focused on combined delivery systems based on nanostructures to administer drugs by the oral cavity, intended for buccal, sublingual, gastric, or intestinal absorption. We analyzed published NP-in-matrix systems and compared main formulation characteristics, pharmacokinetics, release profiles, and physicochemical stability improvements. The reported formulations are mainly semisolid or solid polymers, with polymeric or lipid NPs and one active pharmaceutical ingredient. Regarding drug specifics, most of them are poorly permeable or greatly metabolized. The few studies with pharmacokinetics showed increased drug bioavailability and, sometimes, a controlled release rate. From our knowledge, the gathered data make up the first focused review of these trendy systems, which we believe will help to gain scientific deepness and future advancements in the field.

## Introduction

Medicines administered by the oral cavity have different fates, according to their varied processes: (i) immediate delivery and absorption, (ii) slow delivery from an adherent drug delivery system (DDS), followed by absorption or local action; and (iii) transport to the gastrointestinal region for absorption or local action. The latter is the gold standard for medicines and the most common process used for self-administered drug intake. Recently, buccal permeation (items i and ii) strategies increased in number of developments; this route bypasses hepatic and gastrointestinal effects, which is advantageous to sensitive drugs (Barua et al., 2016).

Regardless of the process, drug solubility can impair efficient release from DDS and subsequent mucosal crossing. Therefore, several solubilization strategies are available, such as salt forms of active molecules, pH modifiers, cosolvents, amorphization, solid dispersions, inclusion complexes, microemulsions, and nanotechnology (Kalepu and Nekkanti, 2015). The latter represents a large portion of published research but a few marketed products and no buccal-based options.

**Figure 1:**
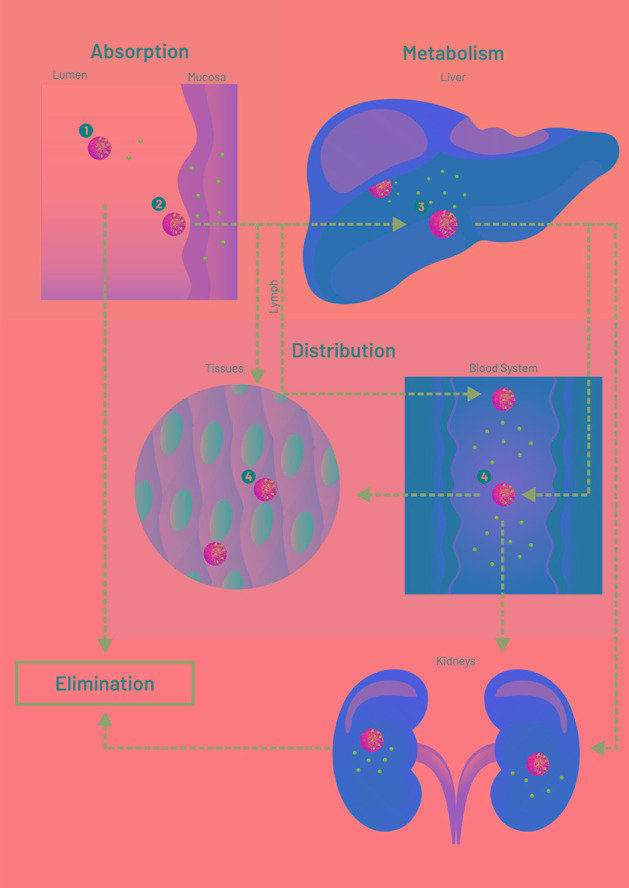
Scheme (not to scale) of drug (blue circles) loaded nanoparticles (NPs, yellow circles) interacting with physiological environments. Nanoparticle modes to enhance drug systemic delivery through the oral or buccal routes. 1: Lumen release (solubility enhancement), followed by NP excretion or degradation. 2: Adherence to mucus or mucosal surface for enhanced drug absorption (no NP permeation), followed by NP excretion or degradation. 3: NP absorption for enhanced plasma half-life, followed by NP liver degradation or lymphatic drainage. 4: Tissue or cell targeting. Figure created for this review in CorelDRAW Graphics Suite X7.

In addition to the solubility enhancement, nanoparticles (NPs) aid drug efficacy in at least four different ways (**Figure 1**). The simpler way relates to transport throughout the gastrointestinal lumen and local release (mode 1). Another possibility consists of carrier adherence to the mucosal surface/mucus to enhance drug absorption, but without carrier crossing (mode 2). Nanoparticles may also be absorbed and transport the drug systemically, enhancing its plasma half-life (mode 3). Absorbed NPs can then perform passive targeting due to charge, size, morphology, and constituents. For a detailed review on mechanisms of NP/absorption and uptake, see Griffin et al. (2016). Mode 4 happens when specific surface molecules or pH-responsive mechanisms allow the carrier to perform an active targeting after absorption. Modes 3 and 4 can still avoid first-pass metabolism if absorption happens in the buccal mucosa or lymphatic vessels. However, like other delivery strategies, NPs can fail to liberate drugs at an expected rate or target, as described in the following paragraphs.

For drug uptake enhancement, NPs need to interact with the respective mucosal surface/mucus during an adequate amount of time. Some particles do stick to mucosa under continuous rinsing, such as thiolated chitosan NPs. But, in this case, NPs were freeze dried and applied on top of the mucosa in an *ex vivo* test (Bernkop-Schnürch et al., 2006). Since powder formulations require specific devices or encapsulation for a precise dosage, they generally require an additional delivery strategy to be administered.

A noteworthy NP disadvantage is the burst effect: a rapid initial drug release followed by a sustained or controlled release. Rapid plasma peaks or local drug concentration are desirable in some therapies; however, burst behavior is frequently uncontrollable and irreproducible. The phenomenon occurs due to several reasons, including weakly bound drugs, molecule migration to the particle surface, and nanomatrix heterogeneity (Kamaly et al., 2016). Strategies to prevent it include coating or nanomatrix reformulation (Kamaly et al., 2016), not always straightforward because both processes change NP physicochemical characteristics.

The majority of oral DDSs are biodegradable, so they will erode or degrade at some point. In fact, some systems rely on both properties as a release mechanism. If the carrier liberates as desired, it may not be resistant to pH/enzymes during traffic or at the action site. An earlier instability may interfere with the desired drug liberation profile, such as for regular gelatin NPs: their degradation in the stomach requires coating, chemical modification or an outermatrix embedment to allow intestinal release (Imperiale and Sosnik, 2013).

Apart from degradation, NPs can change their fate, uptake, or release rate due to *in vitro* or *in vivo* aggregation. The *in vitro* facet shows that cell media for cell culture is quite different from lumen or saliva environments, which makes it hard to predict aggregation during *in vivo* absorption (Moore et al., 2015).

**Figure 2:**
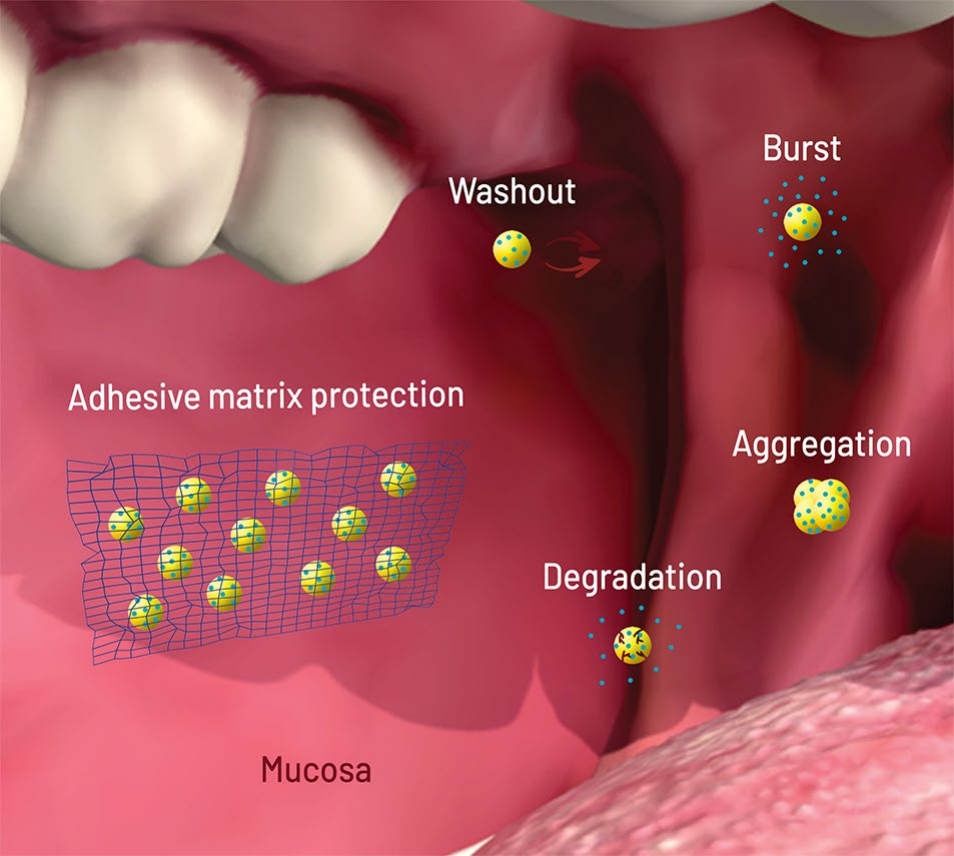
Scheme (not to scale) of the failure modes of nanoparticles (NPs, yellow circles) loaded with drug (blue circles) after oral/buccal administration: early washout in mouth or intestinal cavities; early degradation due to pH or enzymes; burst release upon contact with aqueous media (saliva, stomach acid); aggregation due to pH, osmotic environment, and protein binding. The network on the left shows matrix protection through adhesion (wash out elimination), shielding (degradation and burst elimination/decrease), and physical separation (aggregation elimination). Figure created for this review in CorelDRAW Graphics Suite X7 and Adobe Photoshop CS6.

All failure modes described above can be solved or attenuated with NP dispersion in micromatrices and macromatrices (**Figure 2**), reasons why this technique became important for buccal and oral delivery. As opposed to coating and core modifications, matrix embedment brings an extra added value: it can be applied to several kinds of NPs, with a better general prediction of the release behavior.

Considering all points above, this review intends to analyze pharmacokinetic improvements with the use of NPs incorporated in matrices for drug delivery (NP-in-matrix). Therefore, we focused here on pharmacokinetic data from animal and humans (when available). Several NP-in-matrix systems were tested only with *in vitro* assays. However, correlation of *in vivo* behavior is not guaranteed, and an extensive array of protocols impairs model comparisons (discussed in the following topic). Combined systems resulting in nanosized carriers (NP-in-NP) were not included in this work. Based on the theoretical analysis, we intend to verify and clarify matrix role on permeation and bioavailability of nanocarriers.

## Pharmacokinetics and Evaluation Modes for Oral and Buccal Administration

Pharmacokinetics describes absorption, distribution, metabolism, and excretion processes after drug intake. This knowledge remains essential for safety and efficacy assessments of the therapy in the search for the optimal DDS. However, *in vivo* tests must be a final step toward product development. It should succeed an extensive physicochemical characterization, together with promising *in vitro* data assessing biorelevant properties. **Table 1** lists the most common assays used to evaluate and predict *in vivo* parameters related to pharmacokinetics, together with drug and NP predictors. Because matrix-based formulations aid mainly in absorption, this process will be discussed in more detail below.

**Table 1:** Pharmacokinetic parameters and evaluation strategies for oral/buccal medicines.

	*In vivo* *^a^*	Drug predictors	NP predictors	*In vitro* assays/*ex vivo* assays
Absorption	T max, AUC, *C*_max_	Solubility, lipophilicity (log P), molecular weight, number of hydrogen bond donor groups (Lipinski et al., 2001), log D, polar surface area and pKa (Berben et al., 2018)^b^	Charge, size, bioadhesiveness, lipophilicity, surface modification (Griffin et al., 2016)	Parallel artificial membrane permeation assay (PAMPA) and derivatives (Berben et al., 2018), cell based assays (mainly Caco-2), USP dissolution methods/derivatives^c^, permeation through mucosal tissue (pig, human)
Distribution	Volume of drug distribution at steady state, tissue- plasma partition coefficients	logP, pKa	Targeting ligands	Plasma protein binding assay, *ex vivo* tissue distribution
Metabolism	Metabolites in blood, urine, feces	ligand for liver enzymes, ^d^lipophilicity	Targeting ligands, size, charge, lipophilicity, surface modification (Griffin et al., 2016)	Metabolic activity of hepatocytes, cytochrome P450 (CYP) inhibition assay, liver and intestinal microsome stability
Excretion	Drug in urine, feces	Molecular weight, lipophilicity, pKa	Size	Plasma protein binding

^a^In vivo models include generally mice, rat, rabbit, dog, or monkey species. Monkeys are considered more reliable than other animal species to infer human pharmacokinetic profiles (Furukawa et al., 2014). ^b^Drugs that are substrates for biological transporters do not present predictive permeability with only these parameters. ^c^USP dissolution methods vary depending on the dosage form. They can be predictive for drug with dissolution rate-limited absorption, but complex release kinetics and NP environmental changes may increase in vivo correlation failure. ^d^To check ligand properties, the most common method is molecular docking (Meng et al., 2011).

Nanoparticle permeation happens only after mucus barrier crossing (gastrointestinal) and subsequent cellular uptake for transcytosis (M cells, enterocytes) or paracellular transport. *In vivo* absorption is generally better predicted by *ex vivo* mucosal permeation than *in vitro* tests with synthetic membranes; the extracted tissue offers a biochemical, anatomical, and structural resemblance to its *in vivo* counterpart that is difficult to replicate (Berben et al., 2018).

When it comes to gastrointestinal absorption, Caco-2 cell model stands out among the options, joining cell layer with synthetic membrane permeation. This system is frequently used to classify drugs in a permeation rank to direct dosage form development. However, a review covering Caco-2 correlation with *in vivo* intestinal permeability stressed the test fragility. Based on several studies, the authors concluded that the model is applicable to hydrophobic drugs but fails to predict hydrophilic molecule absorption. Even with hydrophobic drugs, variability is high yet minimized with internal standards. The paracellular route and active transport seem to correlate with higher variability and *in vivo–in vitro* lack of correlation (Larregieu and Benet, 2013).

Regardless of the *in vitro–ex vivo* model, most of them do not promote predigestion of samples. One cannot estimate the effect of gastrointestinal fluids upon NPs without this assessment. Even nondegradable polystyrene NPs (unmodified, aminated, or carboxylated) promoted different *ex vivo* permeation profiles with predigestion, showing its relevance for *in vivo* prediction (Westerhout et al., 2017).

Drug/NP *in vivo* absorption can be estimated by plasma detection if metabolism does not influence their integrity. *In vivo* pharmacokinetics focuses on blood sampling and drug quantification at several time points, generally with rats, rabbits, or humans. The most common parameters include the amount of drug detected over time (area under the curve [AUC]), the highest drug level detected (*C*_max_), and the time the latter happened (*t*_max_). On the other hand, NPs are seldom classified based on plasma levels. Nanoparticle tracking relies mainly on marked particles with fluorescent dyes to observe biodistribution in tissues. An important reminder is that plasma levels do not discriminate buccal from gastric or intestinal absorption.

## NP-in-Matrix DDSs

The following items present a discussion of all articles we could find with *in vivo* pharmacokinetic data of NP-in-matrix DDSs. The NP type directed subdivisions based on their main constituent. Exact values and formulation details are presented in **Table 2**.

**Table 2:** Pharmacokinetic data for NP-in-matrix systems.

References	Matrix type	Matrix excipients	NP-Type	NP excipients	Drug	Pharmacokinetics
(Al-Dhubiab et al., 2015)	Film	HPMC K15 + Carbopol 974P + Eudragit^®^ RL 100 + ethyl cellulose + PEG 200	Polymeric (nanospheres)	PLGA	Acyclovir	• Model: male white rabbits. • Administration: NP-in-buccal film (1 cm^2^) was wetted and applied to the buccal mucosa for 4 h; control animals received oral drug solution (1 mg dose, 1 mL). • Data for control (oral solution): *C*_max_ = 91.61 (ng/mL); *t*_max_ = 2 h; AUC_∞_ = 395.21 ng·h/mL. • Data for buccal combined system (A3): *C*_max_ = 306.04 (ng/mL); *t*_max_ = 6 h; AUC_∞_ = 3116.21 ng·h/mL.
(Nekkanti et al., 2009)**	Tablet	Mannitol	Nanocrystal	HPMC	Cardesan	• Model: male Wistar rats. •Administration: oral suspensions of spray dried drug microparticles or drug nanoparticles at a 10 mg/kg dose. • Data for drug microparticles: *C*_max_ = 0.16 ± 0.10 µg/mL; *t*_max_ = 1.81 ± 1,13 h; AUC_∞_ = 0.31 ± 0.07 ng·h/mL; • Data for drug nanoparticles: *C*_max_ = 0.09 ± 0.03 µg/mL *t*_max_ = 1.06 ± 0.38 h AUC_∞_ = 0.78 ± 0.22 µg·h/mL.
(Rana and Murthy 2013)	Patch (triple layer patch)	HPMC + carbopol + ethyl cellulose	Nanocrystals	PVA	Carvedilol	• Model: rabbits • Administration: NP-in-film (combined system) in a 250 µg/kg dose and oral tablet (control) in a 1 mg/kg dose. • Data for control: *C*_max_ = 48.73 ± 14.1 ng/mL; *t*_max_ = 2 h; AUC_∞_ = 1813.70 ± 42.53 ng·h/mL. • Data for combined system: *C*_max_ = 356.91 ± 29.5 ng/mL; *t*_max_ = 4 h; AUC_∞_ = 4154.37 ± 80.22 ng·h/mL.
(Garhy et al., 2018)	Gel	Sodium carboxymethyl cellulose + hydroxypropyl methylcellulose K4M	Polymeric (nanospheres)	Sodium alginate + Eudragit^®^ RS100	Carvedilol	• Model: male New Zealand rabbits. • Administration: NP-in-buccal gel (3.125 mg of drug) was applied to the mucosa; control animals received equivalent oral dose of commercial tablet. • Data for FG1 (4% HPMC K4M): *C*_max_ = 102.27 ng/mL *t*_max_ = 1h AUC0-∞ = 639.38 ng·h/mL Relative bioavailability = 130.3% increase compared to the market product
						• Data for FG2 (4% HPMC K4M + 2% sodium carboxymethylcellulose): *C*_max_ = 187.41 ng/mL *t*_max_ = 1h AUC0-∞ = 830.59 ng·h/mL Relative bioavailability = 130.3% increase compared to the market product • Data for control (commercial tablet): *t*_max_ = 1.5 h Exact values for *C*_max_ and AUC are not available.
(Rao et al., 2015)	Lipidic (microparticles)	Labrasol^®^	Inorganic nanoparticles	Silica	Cinnarizine	• Model: male Sprague-Dawley rats • Administration: one of four formulations at 10 mg/kg *via* oral gavage (unformulated cinnarizine; silica–lipid hybrid (SLH); pluronic-functionalized silica–lipid hybrid (PLU-SLH); NP-in-microparticle structure and PLU&SLH physical mixture). • Unformulated cinnarizine: *C*_max_ = 262 ± 41 ng/mL; *t*_max_ = 0.6 ± 0.1 h; AUC_∞_ = 656 ± 64 ng·h/mL • SLH: *C*_max_ = 258 ± 23 ng/mL; *t*_max_ = 1.0 ± 0.0 h; AUC_∞_ = 859 ± 133 ng·h/mL • PLU-SLH: *C*_max_ = 427 ± 5 ng/mL; *t*_max_ = 1.3 ± 0.3 h; AUC0-*α* = 1400± 135 ng·h/mL • PLU&SHL: *C*_max_ = 326 ± 39 ng/mL; *t*_max_ = 1.1 ± 0.3 h; AUC_∞_ = 971 ± 161 ng·h/mL
(Lv et al., 2015)	Film	Carboxymethyl chitosan (CCS)	Micelle	Phospholipid and bile salts	Cucurbitacin B (Cu B)	• Model: healthy male rabbits. •Administration: films applied to buccal mucosa. CCS-films containing Cu B loaded phospholipid–sodium deoxycholate–mixed micelles (PL-SDC-MMs), conventional CCS (C-CCS) films and the conventional tablet (Hulusupian), 1 mg/kg. The tablet was administered as oral suspension. • Data for micelles-in-CCS films: *C*_max_ = 2.04 ± 0.50 (µg/mL); *t*_max_ = 5.03 ± 0.34 h; AUC0–36 = 46.43 ± 5.11 (µg·h/mL). • Data for CCS films: *C*_max_ = 0.52 0.37 ± (µg/mL); *t*_max_ ∼7.96 ± 0.97 h AUC0–36 = 4.44 1.21 ± (µg·h/mL). • Data for Cu B marked tablet: *C*_max_ = 3.08 ± 0.52 (µg/mL); *t*_max_ = 3.01 ± 0.82 h; AUC0–36 = 17.23 ± 3.43 (µg·h/mL).
(Lv et al., 2014)	Fast-dissolving oral films (FDOFs)	Pullulan and PEG 400	Micelle	Phospholipid (PL) and bile salts (sodium deoxycholate (SDC))	Cucurbitacin B (Cu B)	• Model: male Wistar rats. • Administration: Cu B-PL/SDC-MMs, the FDOFs containing Cu B-PL/SDC-MMs, and the Cu B suspension, all corresponding to a dose of 2 mg/kg. • Data for Cu B suspension: *C*_max_ = 3.23 ± 0.64 (µg/mL); *t*_max_ = 3.01 ± 0.44 h; AUC(0–24) = 17.13 ± 3.54 (µg·h/mL). • Data for Cu B-PL/SDC-MMs: *C*_max_ = 7.18 ± 1.08 (µg/mL); *t*_max_ = 0.69 ± 0.32 h; AUC(0–24) = 42.25 ± 5.91 (µg·h/mL). • Data for Cu B-PL/SDC-MMs-in-FDOFs: *C*_max_ = 7.82 ± 1.21 (µg/mL); *t*_max_ = 0.67 ± 0.28 h; AUC(0–24) = 45.11 ± 6.13 (µg·h/mL).
(Augustine et al., 2018)*	Microparticle	Alginate + chitosan	Nanosuspension	Alginate	Darunavir/ritonavir	• Model: albine Sprague-Dawley rats. • Administration: 25 mg/kg dose of daruvanir + ritovanir. Groups: unformulated drug, the nanonized drug and the NP-in-microparticle loaded drug (NiMDS). • Data for the unformulated drug: *C*_max_ = 0.14 µg/mL; *t*_max_ = 2.63 h; AUC_last_ = 1.17 µg·h/mL; • Data for nanonized drug: *C*_max_ = 0.11 µg/mL; *t*_max_ = 1.75 h; AUC_last_ = 1.35 µg·h/mL; • Data for NiMDS: *C*_max_ = 0.38 µg/mL; *t*_max_ = 2.75 h; AUC_last_ = 2.67 µg·h/mL.
(Kevadiya et al., 2018)	Oral strip films (OSFs)	HPMC	Nanocrystals	SDS and HPMC	Fenofibrate (FNB)	• Model: New Zealand white rabbits. • Administration: orally/buccal dose equivalent of 30 mg/kg of FNB. Groups: marketed formulation (oral suspension); pristine FNB (suspension); OSFs (5.28 mg/cm^2^ of FNB-NC-D). • Tricor: *C*_max_ = 23.1 ± 8.8 µg/mL; *t*_max_ = 8.0 ± 3.0 h; AUC_∞_ = 654.6 ± 251 µg·h/mL. • FNB: *C*_max_ = 16.8 ± 12.2 µg/mL; *t*_max_ = 8.0 ± 5.8 h; AUC_∞_ = 514.8 ± 374 µg·h/mL. • FNB-NC-D: *C*_max_ = 37.6 ± 10.6 µg/mL; *t*_max_ = 6.0 ± 1.7 h; AUC_∞_ = 931.2 ± 263 µg·h/mL.
(Ahmed et al., 2018)**	Lyophilized tablet (LT)	HPMC, mannitol, silica, Avicel, and plasdone XL	Self-nanoemulsion (SNE)	Anise oil; Tween 80; cosurfactant (methanol; ethanol; propanol; butanol)	Finasteride (FSD)	• Model: healthy male volunteers. • Administration: group I: FSD-SNELTs; group II: FSD-LTs; group III: FSD-marketed tablets (Proscar^®^), containing 5 mg of FSD. • Data for FSD-SNELTs: *C*_max_ = 44.635 ± 3.259 (ng/mL); *t*_max_ = 1.5 ± 0.289 h; AUC_last_ = 721.662 ± 55.085 (ng·h/mL). • Data for FSD-LTs: *C*_max_ = 37.794 ± 1.405 (ng/mL); *t*_max_ = 2 ± 0 h; AUC_last_ = 444.08 ± 37.283 (ng·h/mL). • Data for marketed tablets: *C*_max_ = 29.150 ± 4.798 (ng/mL); *t*_max_ = 3 ± 1 h; AUC_last_ = 487.639 ± 42.989 (ng·h/mL).
(Imperiale et al., 2015)*	Polymeric (microspheres)	Alginate + chitosan coated or not with Eudragit	Nanocrystal	Not mentioned	Indinavir	• Model: mongrel dogs. • Administration: a single dose of 10 mg/kg of: indinavir (IDV) free base, pure IDV nanoparticles and NP-in-microparticle delivery system (NiMDSs); all encapsulated within gastro-resistant capsules. • IDV base: *C*_max_ = 0.34 µg/mL; *t*_max_ = 1.10 h; AUC_∞_ = 0.83 µg·h/mL; • IDV nanoparticles: *C*_max_ = 1.41 µg/mL; *t*_max_ = 2.00 h; AUC_∞_ = 18.16 µg·h/mL; • NiMDSs: *C*_max_ = 0.50 µg/mL; *t*_max_ = 1.80 h; AUC_∞_ = 39.23 µg·h/mL.
(Nidhi et al., 2016)	Patch (transmucosal patch [TP])	Hydroxypropyl cellulose-LF (HPC-LF)	Lipidic (SLN)	Glyceryl palmitostearate + glyceryl monostearate	Lignocaine (Lig) + diclofenac diethylamine (DDEA)	• Model: white New Zealand male rabbits. • Administration: group 1: TP (0.5×0.5 cm) containing lignocaine base (LB) (0.872 mg/kg) and DDEA (1.6248 mg/kg) placed over the anterior mandibular gingiva for 12 hours. Group 2 (control): marketed Lig HCl gel (1.008 mg/kg) over the anterior mandibular gingiva and marketed diclofenac (Dic) sodium administered orally as 1.4 mg/kg. In both groups, the dose maintained was 0.872 mg/kg of Lig and 1.3033 mg/kg of Dic. • Data for TP: - Gingival crevicular fluid (GCF): *C*_max_ = 0.4428 ± 0.28 (µg/mL) (Lig) and 4.6213 ± 0.21 (µg/mL) (Dic); *t*_max_ = 0.25 h (Lig) and 2 h (Dic); AUC_∞_ = 1.6396 ± 1.02 µg·h/mL (Lig) and 808.835 ± 3.25 µg·h/mL (Dic). - Plasma: *C*_max_ = 0.3259 ± 0.03 (µg/mL) (Lig) and 0.1910 ± 0.02 (µg/mL) (Dic); *t*_max_ = 1 h (Lig) and 2 h (Dic); AUC_∞_ = 5.6502 ± 0.72 µg·h/mL (Lig) and 4.1754 ± 0.38 µg·h/mL (Dic).
						• Data for control: - Gingival crevicular fluid (GCF): *C*_max_ = 0.0499 ± 0.02 (µg/mL) (Lig) and 1.6495 ± 0.05 (µg/mL) (Dic); *t*_max_ = 0.5 h (Lig) and 2 h (Dic); AUC_∞_ = 0.1024 ± 0.31 µg·h/mL (Lig) and 8.6204 ± 0.22 µg·h/mL (Dic). - Plasma: *C*_max_ = 0.1645 ± 0.01 (µg/mL) (Lig) and 1.4181 ± 0.15 (µg/mL) (Dic); *t*_max_ = 0.5 h (Lig) and 2 h (Dic); AUC_∞_ = 0.6568 ± 0.01 µg·h/mL (Lig) and 4.6808 ± 0.26 µg·h/mL (Dic).
(Guo et al., 2015)**	Tablet	Not mentioned	Nanocrystals	Pluronic F68, HPMC K4M, HPMC E5, PVP K30	Rebamipide (REB)	• Model: male Sprague-Dawley rats • The test preparation rebamipide nanocrystal tablets (REB-NTs) were compared with that of a reference formulation of Mucosta^®^ tablets (REB-MTs). Dose = 10 mg/kg. • REB-Nts: *C*_max_ = 543.4 ± 150.5 ng/mL; *t*_max_ = 1.67 ± 0.41 h; AUC_∞_ = 2622.3 ± 462.8 ng·min/mL. • REB-Mts: *C*_max_ = 281.5 ± 66.1 ng/mL; *t*_max_ = 1.08 ± 1.34 h; AUC_∞_ = 1187.4 ± 411.8 ng·min/mL.
(Salem et al., 2018)**	Tablet	Nano-silica, microcrystalline cellulose and croscarmellose sodium	Self-nanoemulsion	Surfactants (Tween 80 and Cremophore RH 40), oils (oleic acid, labrafac, labrafil) and cosurfactant (propylene glycol)	Rosuvastatin	• The study was performed in healthy male volunteers. • Administration: SNE-tablet and Crestor^®^; the tablets were administered orally at a dose of 10 mg each. • Data for Crestor^®^: *C*_max_ = 23 885 (ng/mL); *t*_max_ = 3 h; AUC_last_ = 264 210 (ng·h/mL). • Data for SNE tablet: *C*_max_ = 66 521 (ng/mL); *t*_max_ = 2 h; AUC_last_ = 648 219 (ng·h/mL).
(Al-Dhubiab, 2016a)	Buccal Film	HPMC K15 + Carbopol 971P + Eudragit^®^ RS 100 + ethyl cellulose + PEG 400	Polymeric (nanospheres)	PLGA/PVA	Selegiline	• Model: male white rabbits. • Administration: NP-in-buccal film (1 cm^2^) was wetted and applied to the buccal mucosa for 4 h; control animals received oral drug solution (1 mg dose, 1 mL). • Data for control (oral drug solution): *C*_max_ = 264.28 (ng/mL); *t*_max_ = 1 h; AUC_∞_ = 877.07 ng·h/mL. • Data for buccal film (F3): *C*_max_ = 426.58 (ng/mL); *t*_max_ = 4 h; AUC_∞_ = 2935.65 ng·h/mL.
(El-Say et al., 2017)**	Lyophilized tablet (LT)	Porous fumed silica, lactose, and microcrystalline cellulose (Avicel)	Self-nanoemulsion (SNE)	Labrasol, and Transcutol	Vitamin K	• Model: male human volunteers. •Administration: subjects were classified into 3 groups (6 per each). Group I: vitamin K-in-SNELTs; group II: commercial tablet; group III: injected drug ampoule intramuscularly. All groups = 10 mg. • Data for vit K-in-SNELTs: *C*_max_ = 1572.37 ± 120.2 (ng/mL); *t*_max_ = 2.5 ± 0.0 h; AUC_last_ = 7283.34 ± 85.4 (ng·h/mL). • Data for commercial tablet: *C*_max_ = 1054.97 ± 91.04 (ng/mL); *t*_max_ = 3.0 ± 0.0 h; AUC_last_ = 4379.59 ± 202.3 (ng·h/mL). • Data for intramuscular drug ampoule: *C*_max_ = 1868.28 ± 89.2 (ng/mL); *t*_max_ = 2.0 ± 0.0h; AUC_last_ = 8379.76 ± 1434.7 (ng·h/mL).
(Al-Dhubiab, 2016b)	Film	HPMC K100 + Eudragit^®^ RL 100 + Carbopol 974P	Polymeric (nanospheres)	PLGA	Zolpidem	• Model: male white rabbits. • Administration: NP-in-buccal film (1 cm^2^) was wetted and applied to the buccal mucosa for 4 h; control animals received oral drug solution (1 mg dose, 1 mL). • Data for control (oral drug solution): *C*_max_ = 32.34 (ng/mL); *t*_max_ = 1 h; AUC_∞_ = 136.06 ng·h/mL. • Data for buccal film (Z4): *C*_max_ = 52.54 (ng/mL); *t*_max_ = 1.5 h; AUC_∞_ = 236.00 ng·h/mL.
(Tong et al., 2018)**	Tablets	Anhydrous dibasic calcium phosphate (Fujicalin^®^)	Self-nanoemulsion (SNE)	Soybean lecithin and glycocholic acid (surfactant) and Transcutol HP (cosurfactant)	Vitamin K1 (VK1)	• Model: beagle dogs. •Administration: six healthy beagle dogs randomly divided into two groups (SNE-L tablets and conventional VK1 tablets), receiving a single 10 mg oral dose.
						• Data for combined system: *C*_max_ = 575.46 ± 84.27 (ng/mL); *t*_max_ = 1.67 ± 0.58 h; AUC_∞_ = 1716.33 ± 264.20 (ng·h/mL). • Data for control (commercial tablet): *C*_max_ = 249.23 ± 79.05 (ng/mL); *t*_max_ = 2.0 ± 1.0 h; AUC_∞_ = 866.14 ± 215.45 (ng·h/mL).
(Attili-Qadri et al., 2013)	Microparticle	Eudragit L + HPMC	Polymeric (nanocapsule)	Glyceryl tributyrate + oleoyl polyoxylglycerides + PLGA	Docetaxel	• Model: minipigs. • Administration: oral dose of Taxotere (commercial) and combined system (1.25 mg/kg).
						• Data for control (oral solution of commercial drug): *C*_max_ = 97.6 (ng/mL); AUC_∞_ = 797.7 ng·h/mL. • Data for combined system: *C*_max_ = 817.9 (ng/mL); AUC_∞_ = 7,923.1 ng·h/mL.
(Nassar et al., 2011)*	Microparticle	Eudragit L + HPMC	Polymeric (nanocapsule)	Glyceryl tributyrate + oleoyl polyoxylglycerides + PLGA	Docetaxel	• Model: Sprague-Dawley male rats • Administration: intravenous dose of drug-loaded NPs and oral dose of combined system (5mg/kg). • Data for drug-loaded NPs: AUC = 1,441.9 ng·h/mL. • Data for oral combined system: AUC = 5,754.5 ng·h/mL
(Soudry-Kochavi et al., 2015)**	Microparticle	Eudragit L + HPMC	Polymeric (nanosphere)	BSA + dextran + sodium trimetaphosphate	Exenatide	• Model: Sprague-Dawley male rats. • Administration: drug solution and commercial drug (Byetta™) were administered subcutaneously (SC)(65µg/kg). Combined systems DX-50 and DX-150 were administered orally (by gavage) (165 µg/kg). • Data for control (SC commercial drug): *C*_max_ = 1.22 µg/mL *t*_max_ = 1.33 h AUC = 31.01 h·µg·10^-2^/mL. • Data for control (SC drug solution): *C*_max_ = 1.06 µg/mL *t*_max_ = 0.83 h AUC = 26.37 h·µg·10^-2^/mL. • Data for oral combined system DX-50 (50 mg dextran): *C*_max_ = 2.09 µg/mL *t*_max_ = 1.33 h AUC = 59.45 h·µg·10^-2^/mL. • Data for oral combined system DX-150 (150 mg dextran): *C*_max_ = 1.80 µg/mL *t*_max_ = 1.00 h AUC = 36.00 h·µg·10^-2^/mL.

NP, nanoparticle; AUC, area under the curve that describe drug concentration over time, measured in plasma; C_max_, the maximum drug concentration measured in plasma; t_max_, the time taken to reach the maximum drug concentration; PVA, polyvinyl alcohol; PLGA, poly(lactic-co-glycolic acid); HPMC, hydroxypropyl methylcellulose; PEG, polyethylene glycol; SDS, sodium dodecyl sulfate; PVP, polyvinylpyrrolidone; BSA, bovine serum albumin.

*Studies comparing pharmacokinetic data for NP-in-matrix and free NPs.

**NP-in-matrix systems for oral delivery.

### Polymeric NPs

Polymers vary on their degradation, polarity, source, and chain size properties (Hallan et al., 2016). Polymeric based NPs present scalable manufacturing methods and capability to load a wide range of drug types (Crucho and Barros, 2017). The most common natural polymers for NPs are chitosan, sodium alginate, dextran, gelatin, and albumin. These hydrophilic proteins and carbohydrates degrade in physiological conditions, besides their biocompatibility, which helps to avoid side effects (Boateng and Areago, 2014; Madkhali et al., 2019; Bronze-Uhle et al., 2017; Kim et al., 2001). Alginate and chitosan still exhibit bioadhesive nature, which increases NP efficacy for mucosal delivery (Boateng and Areago, 2014). In turn, albumin discharges drugs *via* desorption without significant burst effects (Jiang and Stenzel, 2016). We have not found any pharmacokinetics data for NP-in-matrix systems based on gelatin or chitosan NPs; however, several studies discussed in this article address these excipients as matrix components.

Concerning alginate NPs, Garhy et al. loaded carvedilol in these carriers and further incorporated this system in buccoadhesive gels. The *in vitro* release assay showed burst behavior for all 12 different NP formulations. However, NP-in-gel formulations (FG1 and FG2) indicated that the gelling agent delayed carvedilol release, which diminished burst. FG1 gel contained hydroxypropyl methylcellulose (HPMC), whereas le FG2 presented the same HPMC concentration and sodium carboxymethylcellulose. Rabbits treated with FG2 formulation showed a two-fold increase in relative bioavailability compared to the market product. The increase in bioavailability occurred probably due to NP enhancement in drug solubility and the buccal bypass of the first-pass effect (Garhy et al., 2018).

Albumin properties were tested in exenatide-loaded bovine serum albumin/dextran NPs; the peptide was adsorbed to the carrier protein and released in a sustained manner due to dextran crosslinkings. This loaded NP was incorporated in gastroresistant microparticles of Eudragit L/HPMC. The incorporation led to macromolecule protection and decreased release rates of the peptide cargo. Consequently, pharmacokinetic data showed that NP-in-matrix resulted in a high relative oral bioavailability of 77% compared to a subcutaneous injection of the commercial equivalent medicine (Soudry-Kochavi et al., 2015).

Among the synthetic polymers, the aliphatic polyesters and their copolymers are the most used for drug delivery because of their biodegradability and biocompatibility. One of the most popular is the poly(lactic acid), often combined with glycolide to form the hydrophobic copolymer poly(lactic-co-glycolic acid) (PLGA) (Washington et al., 2017); PLGA exhibit high stability in biological fluids and long clinical experience; it was the only synthetic polymer to compose a nanostructure part of an NP-in-matrix dosage form with pharmacokinetic data. All the drugs entrapped into these studied systems are considered poorly water-soluble drugs.

Three different studies reported buccal films as matrices for PLGA NPs. The incorporated drugs were acyclovir (Al-Dhubiab et al., 2015), selegiline (Al-Dhubiab, 2016a) and zolpidem (Al-Dhubiab, 2016b). *In vitro* studies showed that film formulations prolonged drug liberation in a composition-dependent mode. The results of *ex vivo* studies with rabbit buccal mucosa showed that these nanospheres can permeate the tissue. *In vivo* results confirmed the predictions, whereas male rabbits demonstrated an increased drug bioavailability with the NP-in-film strategy. After incorporating NPs in polymeric films, the bioadhesive properties increased the residence time in oral cavity. It was also observed that *C*_max_, AUC, and *t*_max_ improved with the use of combined systems when compared to controls (oral drug solutions, results in **Table 2**).

The PLGA NP improvements may come as well from a micro matrix strategy. Nassar et al. (Nassar et al., 2011) used docetaxel-loaded PLGA nanocapsules in entero-coated microparticles. The microcarrier released NPs that penetrated the enterocytes of rats, bypassed permeability-glycoprotein pump, and apparently circumvented gut metabolism of the drug. An oral administration of NP-in-matrix resulted in higher bioavailability than intravenous solution of the free drug (commercial formulation, 276%) and its NP formulation (400%). A subsequent study with minipigs confirmed the pattern obtained with rats; the superiority of NP-in-matrix over NPs was attributed to lymphatic transportation that changed drug biodistribution (Attili-Qadri et al., 2013).

### Lipidic NPs

Lipid NPs are well-established DDSs due to their high biocompatibility, biodegradability, low toxicity, and applicability to various administration routes (Chime and Onyishi, 2013; Wissing et al., 2004; Shastri, 2017; Teixeira et al., 2017; Puri et al., 2009). The main lipid NPs reported are liposomes, solid lipid nanoparticles (SLNs), nanostructured lipid carriers, and nanoemulsions. The hydrophobic cores enhance drug solubility and protect it from the environment; the surfactant layer separates particles by steric or electrical hindrance. The exception is the liposome vesicle form, which allows loading of hydrophobic and hydrophilic drug molecules into its outer layer or aqueous core, respectively (Karamanidou et al., 2016). Among the cited particles, only NLC did not present *in vivo* data, probably because it is the most recent development among this group.

Lipidic NPs may deform under mechanical stress, such as tableting; they can also undergo lipid phase transitions upon heating, such as for film casting. Therefore, Hazzah et al. incorporated curcumin SLN in freeze-dried polymeric sponges to avoid the cited issues. Human studies showed that curcumin SLN-in-polycarbophil sponge has higher *C*_max_, *t*_max_, and AUC than the SLN-in-HPMC sponge (**Table 2**). In accordance, polycarbophil formulation adhered to the mucosa for a longer time (15 h, compared to 4 h) and presented higher matrix porosity and homogeneous distribution of SLNs. The decreased porosity of HPMC sponges diminished swelling and consequent interaction with mucin. Also, SLNs remained onto the surface of HPMC sponges, lowering its adhesion property to the oral mucosa and releasing the NPs faster than polycarbophil. Although pure NPs were not tested *in vivo*, *in vitro* release showed that NP-in-sponge eliminated the burst effect of the nanodispersions, which improves prediction of therapy outcomes and avoids possible plasma peaks (Hazzah et al., 2015).

Ahmed et al. also used freeze-dried polymeric matrix (gelatin “tablets,” no compression step) to load lipidic self-nanoemulsions (SNEs) of finasteride. Different from the mucoadhesive sponges, the freeze-dried tablets melt on the mouth upon contact with saliva. These macrocarriers aim as fast release as possible for buccal and gastrointestinal absorption. Both formulated tablets (with or without SNE) presented in human higher *C*_max_ and shorter *t*_max_ of the drug than the marketed tablets. Likewise, AUC and Mean Residence Time (MRT) indicated superiority of NP-in-tablet system. The improved drug bioavailability may enhance the therapeutic effects (Ahmed et al., 2018).

A variety of this technique decreased the low bioavailability of oral vitamin K. The SNE was loaded on porous silica carriers and later incorporated in lyophilized tablets. SNE-in-lyophilized tablets increased absorption rate and extent of vitamin K in humans compared to marketed tablets. Even better, the NP-in-matrix system presented AUC similar to the commercial intramuscular injection (El-Say et al., 2017). Powdered SNE loaded with vitamin K_1_ was also incorporated into regular tablets, but with different vitamin loadings. Because SNEs form nanoparticles upon contact with gastrointestinal fluid, NP deformation was not a concern. Beagle dogs administered with SNE-in-tablet showed a 2.3-fold increase in vitamin K_1_ *C*_max_ and 1.98-fold in AUC (Tong et al., 2018). The increment in bioavailability probably happened due to higher surface area and consequent higher drug dissolution rate in the gastrointestinal tract (Gong et al., 2016).

SNE-in-tablet was also applied to rosuvastatin delivery, increasing solubility and bypassing hepatic first-pass metabolism. Male humans taking SNE-in-tablet had rosuvastatin AUC increased 2.45 times and *C*_max_ increased 2.78 times compared with the intake of commercial rosuvastatin tablets. Similarly, *t*_max_ decreased with drug administration in SNE tablet, highlighting its benefits (Salem et al., 2018).

The latter lipid NP-in-matrix with published *in vivo* pharmacokinetics is the phospholipid-bile salts-mixed micelles. Cucurbitacin B was loaded in these micelles to address its water insolubility, toxicity, and gastrointestinal side effects. The use of fast-dissolving oral films of pullulan as a matrix maintained the *in vivo* absorption properties like micelles and promoted a significant increase in the oral bioavailability of cucurbitacin B in Wistar rats (highest values of *C*_max_ and AUC, lowest *t*_max_), when compared to the free suspension drug. Also, the matrix did not interfere with Cu B-micelles original structure (Lv et al., 2014). These micelles in carboxymethyl chitosan buccal film rendered a mucoadhesive formulation, which released the drug for a longer period. It resulted in 2.69-fold increase in bioavailability in rabbits when compared to marketed tablets and 10.46 times the film formulation without NP. The team reported that buccal mucosa barrier probably explains higher *C*_max_ and lower *t*_max_ of oral tablets; high AUC from film formulations may be owed to the presence of permeation enhancers in it and the nanosized drug, ensuring the increase in the amount of drug penetration into the blood (Lv et al., 2015). It would be interesting to compare the influence of the different matrices in cucurbitacin B kinetics, but the animal models differ in species, drug dose, and period of evaluation.

### Inorganic NPs

Inorganic nanoparticles are flexible carriers that allow surface modification, drug targeting, and modified drug release. This group includes silica, clay, and metals as excipients arranged in nanoparticles, nanotubes, or nanorods/nanoparticles, respectively (Kerdsakundee et al., 2017; Rao et al., 2011; Zhang et al., 2018). The silica-based ones were the only inorganic NP-in-matrix compositions tested *in vivo* for oral administration. Although biocompatible and with a well-defined/modifiable structure, they do not adhere to mucosa and are good candidates for matrix incorporation (Slowing et al., 2008).

To improve the oral delivery of the poorly soluble drug cinnarizine, an antihistamine and calcium-channel blocker, Rao S. et al. developed a pluronic functionalized silica–lipid hybrid microparticle. Pluronic acts as a polymeric precipitation inhibitor, avoiding recrystallization of cinnarizine dissolution; the silica–lipid hybrid microparticle improved drug partition by producing a hydrophobic microenvironment. Bioavailability was compared between the cinnarizine loaded functionalized silica-lipid hybrid system, unformulated cinnarizine and a cinnarizine loaded nonfunctionalized silica-lipid system; the in vivo design included a single dose of 10 mg/kg of each formulation *via* oral gavage. The study resulted in more than 2.1-fold improvement in the AUC and 1.6-fold improvement in *C*_max_ of cinnarizine of the functionalized NP-in-microparticle structure in comparison to the unformulated one and a 1.6-fold improvement in both AUC and *C*_max_ in comparison with the nonfunctionalized formulation, resulting in an overall improved bioavailability of cinnarizine (Rao et al., 2015).

### Drug-Based NPs

Poor solubility, low bioavailability, and short stability *in vivo* are limiting problems in the development and delivery of new active ingredients (Liu et al., 2012). To overcome these issues, one can entrap the drug in a nanocarrier or reduce its particles to obtain a nanosized range, such as nanocrystals. Compared to NPs, drug-based particles offer higher drug loading (nearly 100%) with generally less excipients and uniform/stable physical nature (Liu et al., 2012).

Accordingly, indinavir nanonization increased absorption (from 0.83 to 18.16 µg·h/mL) and *t*_max_ (from 1.10 to 2.50 h) of the free base drug after administration of a single oral dose in mongrel dogs; however, NP-in-microparticle alginate/chitosan particles performed much better than pure nanocrystals (AUC of 39.23 µg·h/mL and *t*_½_ value of 76.3 h). This represented an increase of the oral bioavailability and the apparent *t*_½_ of 47 and 95 times, compared to the free drug. The increment in release time may improve HIV treatment, which demands long-term therapy and frequent dosing (Imperiale et al., 2015). Likewise, darunavir and ritonavir benefited from an NP-in-microparticle oral delivery system for nanocrystals. The difference was that NP-in-microparticle increased the oral bioavailability of the combined drugs by 2.3-fold compared with the NP only and the free drug. In this case, nanonization alone was not able to increase absorption (Augustine et al., 2018).

Nanocrystals vehicled in tablets and buccal films allow easier self-administration, increased dosage precision, and superior performance. Rebamipide presents only 10% of oral bioavailability in humans due to poor solubility, which is the reason Guo and coworkers formulated an NP-in-tablet version of the medicine. Drug nanocrystals were stabilized with HPMC and polyvinylpyrrolidone before tablet incorporation. The relative oral bioavailability of REB nanocrystal tablets was 256.8% in rats (reference Mucosta^®^ tablets) (Guo et al., 2015). Nekkanti’s group also used HPMC-stabilized nanocrystals for candesartan cilexetil delivery, further incorporated in mannitol-based tablets. This prodrug belongs to the low solubility/long-term therapy group, such as rebamipide. *In vivo* studies confirmed dosage form benefits: Wistar rats presented 2.51-fold increase in AUC, a 1.77-fold increase in *C*_max_, and a decreased *t*_max_ (1.81–1.06 h) compared to the free prodrug (Nekkanti et al., 2009).

Rana and Murthy developed a three-layer buccal film: a mucoadhesive layer, a layer containing nanosuspension of carvedilol nanocrystals and a backing membrane. The structure aimed to prevent the first-pass metabolism, raising drug bioavailability. *C*_max_ of the buccal patch was 7.3 times higher than that of the oral tablet, and *t*_max_ exhibited by the patch was 4 h in comparison to 2 h for oral tablet. The NP-in-microparticle structured buccal patch has been designed as a novel platform for potential buccal delivery of drugs having high first-pass metabolism (Rana and Murthy, 2013).

Another application of nanosized drugs is the development of better suitable dosage forms of existing drugs to attend the patient needs. Kevadiya et al. developed an oral strip-film containing nanocrystals of the cholesterol-reducing agent fenofibrate, a very low solubility prodrug, aiming to create a fast disintegrating solid dosage form ideal for emergency administration and for patients with swallowing difficulties. New Zealand white rabbits were divided in three groups: the first received the oral commercial formulation Tricor in suspension form; the second received the suspension of fenofibrate nanocrystals (pristine FNB) and the third received the oral striped-films containing fenofibrate nanocrystals (OSF). Pharmacokinetics data showed a higher *C*_max_ and lower *t*_max_ of OSF when compared to the marketed Tricor formulation and pristine FNB. The AUC was also higher for OSF formulation (931.26 µg·h/mL) compared to Tricor (654.6 ± 251 µg·h/mL) and pristine FNB (514.8 ± 374 µg·h/mL); and the *t*_max_ (h) of OSFs was found to be 2 h earlier than the *t*_max_ of Tricor and pristine FNB (Kevadiya et al., 2018).

## Conclusion

Because most of the articles we gathered date from this decade, NP-in-matrix approach still has a long way for exploitation. In our findings, most matrices belong to the polymeric group, based on classical excipients in the market, like HPMC. However, they vary from buccal to oral delivery, with rapid or slow degradation to offer an immediate or modified release. The use of an external matrix to incorporate nanoparticles brought several advantages to formulations. This type of system reduced burst effect, avoided NP degradation in gastrointestinal tract, increased residence time in the mouth, modulated *t*_max_, and bypassed first-pass metabolism (buccal forms and some oral forms). Thus, the systemic bioavailability of the tested drugs was successfully enhanced.

Buccal release belongs to the trending strategies for drugs, so we expect an increase in pharmacokinetic studies concerning NP-in-matrix buccal delivery. Nanoparticle types will probably expand too, as the actual group is mainly PLGA, nanocrystals, and some lipid particles. In summary, we believe this article compiled several evidences and possible pitfalls of this strategy, which will help future developments on the field.

## Author Contributions

RC: synthetic polymeric NP-in-matrix research and writing. DG— inorganic NP-in-matrix research and writing. VB-d-A: lipidic NP-in-matrix research and writing. JC: tables, figures and conclusion. LO-N: review design, introduction and pharmacokinetics writing, scientific revision.

## Funding

FAPESP 14/14457-5 - article charges funding CAPES 001 - scholarships for graduate students (RF, DG, VB-d-A, JC). This study was financed by the Coordenação de Aperfeiçoamento de Pessoal de Nível Superior - Brasil (CAPES) - Finance Code 001 and FAPESP project 2019/08281-5 and 2019/19696-1.

## Conflict of Interest Statement

The authors declare that the research was conducted in the absence of any commercial or financial relationships that could be construed as a potential conflict of interest.
